# Ready, Set, Go! Low Anticipatory Response during a Dyadic Task in Infants at High Familial Risk for Autism

**DOI:** 10.3389/fpsyg.2016.00721

**Published:** 2016-05-25

**Authors:** Rebecca J. Landa, Joshua L. Haworth, Mary Beth Nebel

**Affiliations:** ^1^Center for Autism and Related Disorders, Kennedy Krieger Institute, BaltimoreMD, USA; ^2^Department of Psychiatry and Behavioral Sciences, Johns Hopkins University School of Medicine, BaltimoreMD, USA; ^3^Center for Neurodevelopmental and Imaging Research, Kennedy Krieger Institute, BaltimoreMD, USA; ^4^Department of Neurology, Johns Hopkins University School of Medicine, BaltimoreMD, USA

**Keywords:** autism, infant, anticipation, social, motor

## Abstract

Children with autism spectrum disorder (ASD) demonstrate a host of motor impairments that may share a common developmental basis with ASD core symptoms. School-age children with ASD exhibit particular difficulty with hand-eye coordination and appear to be less sensitive to visual feedback during motor learning. Sensorimotor deficits are observable as early as 6 months of age in children who later develop ASD; yet the interplay of early motor, visual and social skill development in ASD is not well understood. Integration of visual input with motor output is vital for the formation of internal models of action. Such integration is necessary not only to master a wide range of motor skills, but also to imitate and interpret the actions of others. Thus, closer examination of the early development of visual-motor deficits is of critical importance to ASD. In the present study of infants at high risk (HR) and low risk (LR) for ASD, we examined visual-motor coupling, or action anticipation, during a dynamic, interactive ball-rolling activity. We hypothesized that, compared to LR infants, HR infants would display decreased anticipatory response (perception-guided predictive action) to the approaching ball. We also examined visual attention before and during ball rolling to determine whether attention engagement contributed to differences in anticipation. Results showed that LR and HR infants demonstrated context appropriate looking behavior, both before and during the ball’s trajectory toward them. However, HR infants were less likely to exhibit context appropriate anticipatory motor response to the approaching ball (moving their arm/hand to intercept the ball) than LR infants. This finding did not appear to be driven by differences in motor skill between risk groups at 6 months of age and was extended to show an atypical predictive relationship between anticipatory behavior at 6 months and preference for looking at faces compared to objects at age 14 months in the HR group.

## Introduction

Autism spectrum disorders (ASD) are defined by social communication impairment along with repetitive and restricted patterns of behavior and interests (RRBI) (American Psychiatric Association [APA], 2013). However, motor impairments, beyond repetitive motor mannerisms, are prevalent in ASD, and school age children with ASD demonstrate abnormal patterns of motor learning. When learning novel movements, children with ASD show a bias against visual feedback from the external world in favor of proprioceptive feedback from their own bodies (Haswell et al., 2009). This sensory bias has been replicated several times, appears to be specific to ASD (Izawa et al., 2012) and is a robust predictor of motor, imitation and social skill deficits (Haswell et al., 2009; Izawa et al., 2012; Marko et al., 2015). This reduced sensitivity to visual feedback also is consistent with reports that children with ASD exhibit patterns of motor deficits that reflect impairments in visual-motor integration. Children with ASD perform worse on clinical assessments of visual-motor integration (Mayes and Calhoun, 2007) and struggle to incorporate visual input into movement planning (Glazebrook et al., 2009; Dowd et al., 2012). Children with ASD also display particular difficulty with motor tasks that rely heavily on hand-eye coordination, including ball skills (Siaperas et al., 2012; Whyatt and Craig, 2012; Ament et al., 2015) and gesture imitation (Edwards, 2014). Indeed, in ball catching, for example, children must integrate visual and motor systems, using visual information to anticipate the timing of the ball’s arrival at their body and mount the motor act of preparing to catch the ball before the ball’s actual arrival (Ament et al., 2015). Furthermore, recent neuroimaging evidence suggests that altered connectivity, within and between visual and motor networks, may contribute to motor and social impairments in children with ASD (Fishman et al., 2014; Nebel et al., 2014, 2016).

The combined results of these prior behavioral and imaging studies implicate a connection between visual-motor integration and the severity of core ASD symptoms. However, it is unclear both when these visual-motor integration deficits emerge in the developmental cascade of behavioral and neural abnormalities leading to ASD and when their association with ASD symptomatology can be detected. During the prodromal period of ASD (Landa et al., 2007; Yirmiya and Charman, 2010; Landa et al., 2013), non-specific (to ASD) differences in motor development have been observed in infants at high risk (HR) for ASD (i.e., children who have at least one older sibling with an ASD diagnosis) (Landa and Garrett-Mayer, 2006; Flanagan et al., 2012; Leonard et al., 2014; Libertus et al., 2014). Early motor delays also appear to have predictive value for the later development of social and communication impairments (Bhat et al., 2012; Leonard et al., 2014), including ASD (Sutera et al., 2007; Bolton et al., 2012; Flanagan et al., 2012). Such social deficits are detectable not only during interaction with others, but also in eye tracking paradigms such as those demonstrating atypical attention to faces (Chawarska et al., 2010). Compelling evidence also suggests that atypical visual attention is observable by mid infancy in infants at high familial risk (HR) for ASD (Ibanez et al., 2008; Elsabbagh et al., 2009), including reduced attention to faces in children that are later diagnosed with ASD (Osterling and Dawson, 1994; Maestro et al., 2001).

While promising, these studies have focused on body movements or visual attention in isolation. Focused investigation of the development of visual-motor integration offers unique potential to provide a window into ASD pathophysiology for several reasons. Many of the early behaviors used to identify children with ASD (e.g., joint attention and the manipulation and sharing of objects) require efficient coordination between visual and motor systems. In addition, alterations in early motor and visual attention development could perturb the typical coupling of visual perception and reaching. Such coupling is important for infants to develop an understanding of event sequences (Hauf and Prinz, 2007) and for infants to learn how to plan action in order to influence the outcome of an event (Cattaneo et al., 2007). In infants later diagnosed with ASD, anticipatory abnormality has been reported (Bryson et al., 2007; Brisson et al., 2012). For example, a recent retrospective study provides evidence of reduced anticipatory responses in young children with ASD during feeding (Brisson et al., 2012). Integration of visual input with motor output is vital for the formation of internal models of action necessary to develop a wide range of motor skills and also to imitate (Wolpert et al., 2003) and interpret the actions of others (Falck-Ytter et al., 2006), which could play an important role in interpreting the intentions of others and generating socially appropriate behavior during social interaction (Pfister et al., 2013; Angus et al., 2014). Thus, closer examination of motor, and specifically visual-motor, deficits in infants at HR for ASD is of critical importance.

Examining visual-motor integration in infants at HR for ASD also may reveal important information about the cohering of brain systems. As an infant learns a new action, the brain purportedly constructs an association between motor commands and sensory feedback. These internal models of the action allow the brain to predict the sensory consequences of self-generated motor commands and to produce motor commands that maximize expected reward while minimizing effort (Shadmehr and Krakauer, 2008). The emergence of reaching in infancy offers a unique developmental window within which to investigate trajectories of visual-motor integration in children at HR for ASD. When infants begin to reach, they swat at toys (Thelen et al., 1993) and struggle to adjust their reaching response to anticipate the size, texture or orientation of objects prior to contact (Corbetta et al., 2000). Around the age that motor delays have been observed in HR infants, infants with typical development begin to incorporate visually available information into reaching movements. Typically, 5-month-old infants rely on haptic feedback and align their hands only after making contact with an object; however, by age 8 months, infants begin to use visual feedback to anticipate contact while still reaching (McCarty et al., 2001; Witherington, 2005). Reaching toward a moving object is particularly taxing on visual-motor integration and the perception-action coupling system. To successfully complete the task, a child must attend to and use visual information regarding the speed of the moving object as well as its size and shape to appropriately time and control the catching action (van der Meer et al., 1994, 1995).

In the present study of infants at high and low familial risk for ASD, possible behavioral markers of visual-motor coupling during a naturalistic, interactive dyadic ball rolling activity were investigated. A hypothesis was that, compared to LR infants, HR infants would display decreased anticipatory response (perception-guided predictive action) to a ball rolling toward them. Anticipatory responses require, among other things, visual-motor integration (perception-action coupling) and motor planning, the development of which are hypothesized to be delayed in HR infants based on reported delays in motor and visual attention. Also examined was visual attention before and during ball-rolling, hypothesized to contribute to differences in anticipatory behavior. Given the strong association between visual-motor coupling and the severity of social deficits in school age children with ASD (Haswell et al., 2009; Izawa et al., 2012; Marko et al., 2015), we examined whether maturity of anticipatory response at age 6 months would show an association with maturity in sensorimotor and social (ASD symptomatology and attention to social stimuli – faces) functioning during transition from the prodromal period into the full manifestation of ASD (Landa et al., 2013), age 14 months, in HR and LR groups.

## Materials and Methods

### Participants

The Johns Hopkins Medical Institutional Review Board approved all aspects of this study. Written informed consent was obtained from the legal guardians of all participants prior to enrollment.

Participants included 66 infant siblings of children with ASD (HR) and 43 infants at low risk (LR) for ASD, defined as having no familial history of ASD, who were enrolled in a prospective, longitudinal study focusing on early patterns of development in ASD (Landa and Garrett-Mayer, 2006) and early markers of ASD. The infants in this report represent all those who completed the experimental ball rolling task described below (except for five who completed the task but data were uncodable due to infant fussiness or parent intervention during the task). Demographic information about the sample is provided in **Table 1**. Children in the study are tested at age 6 and 14 months.

**Table 1:** Participant demographics at ages 6 and 14 months.

Measure	Group at age 6 months				Group at age 14 months			
	LR		HR		LR		HR	
	*n* = 43		*n* = 66		*n* = 33		*n* = 53	
lMale/*female*	24/*19*		36/*30*		18/*15*		29/*24*	
lHas (a) Sibling(s)	26		66		20		53	

	** 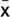 **	***SD***	** 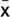 **	***SD***	** 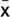 **	***SD***	** 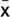 **	***SD***
	
Age	6.7	0.51	6.7	0.56	14.8	0.77	14.8	0.78
AOSI Total	5.4	3.0	7.5^∗^	3.5				
MSEL Gross Motor	6.5	1.0	6.3	1.4	15.7	2.2	14.9	1.8
MSEL Fine Motor	7.1	1.2	6.5^∗^	1.3	17.1	1.4	16.5	1.7
MSEL Visual Reception	7.0	1.0	6.7	0.97	17.2	1.8	15.6^∗^	1.9
ADOS-T overall					3.5	2.2	8.2^∗^	5.6
ADOS-T SA total					2.2	2.0	6.3^∗^	4.8

*Out of 109 infants who completed the ball-rolling experiment at age 6 months, 21 did not complete a 14-month visit. Nine of the 21 attrited from the study and 12 missed their 14-month study visit. AOSI, Autism Observation Scale for Infants; ADOS-T, Autism Diagnostic Observation Schedule-Toddler Module; SA, Social Affect. ^∗^ indicates significant difference between LR and HR groups, *p* < 0.05.*

Out of 109 infants who completed the ball-rolling experiment at age 6 months, 21 did not complete a 14-month visit. Nine of the 21 attrited from the study and 12 missed their 14-month study visit. Fifty-four of the 66 HR infants and 34 of the 43 LR infants who completed the ball-rolling task at age 6 months also completed the Autism Diagnostic Observation Schedule (ADOS; Lord et al., 1999, 2012), the Mullen Scales of Early Learning (MSEL; Mullen, 1995), and an eye tracking task at age 14 months. Two children (one in each group) who received the ADOS-Generic (Lord et al., 1999) Module 1 were removed from further analyses. At age 14 months, the ADOS-Toddler (ADOS-T; Lord et al., 2012) was administered to the remaining 86 children, enabling us to examine the relationship between infants’ anticipatory behavior at age 6 months and social functioning at age 14 months.

Participants were recruited through local Infants and Toddlers programs, ASD advocacy groups, Kennedy Krieger outpatient clinics, pediatricians’ offices, community events and parent self-referral. Inclusion criteria were (1) being 5 months, 0 day to 8 months 30 days and (2) having English as the primary language of the infants’ parents. Infants were excluded if they had any of the following, per review of medical and developmental history: (1) <34 weeks or >42 weeks gestational age; (2) <2300 g birth weight; (3) severe birth trauma; (4) head injury sustained before or during the study; (5) illicit drug or excessive alcohol exposure; (6) major hearing or visual impairment; (7) non-febrile seizures; (8) any known genetic syndrome; or (9) severe birth defects. All infants in the HR group had an older sibling with idiopathic ASD, confirmed by the ADOS, meeting DSM-IV autism or Pervasive Developmental Disorder-Not Otherwise Specified or DSM-5 criteria for ASD (American Psychiatric Association [APA], 1994, 2013), and having a clinical best estimate of ASD established by a trained clinician. Twenty-six of the 43 infants in the LR group had a typically developing older sibling.

### Experimental Ball Rolling Task

**FIGURE 1:**
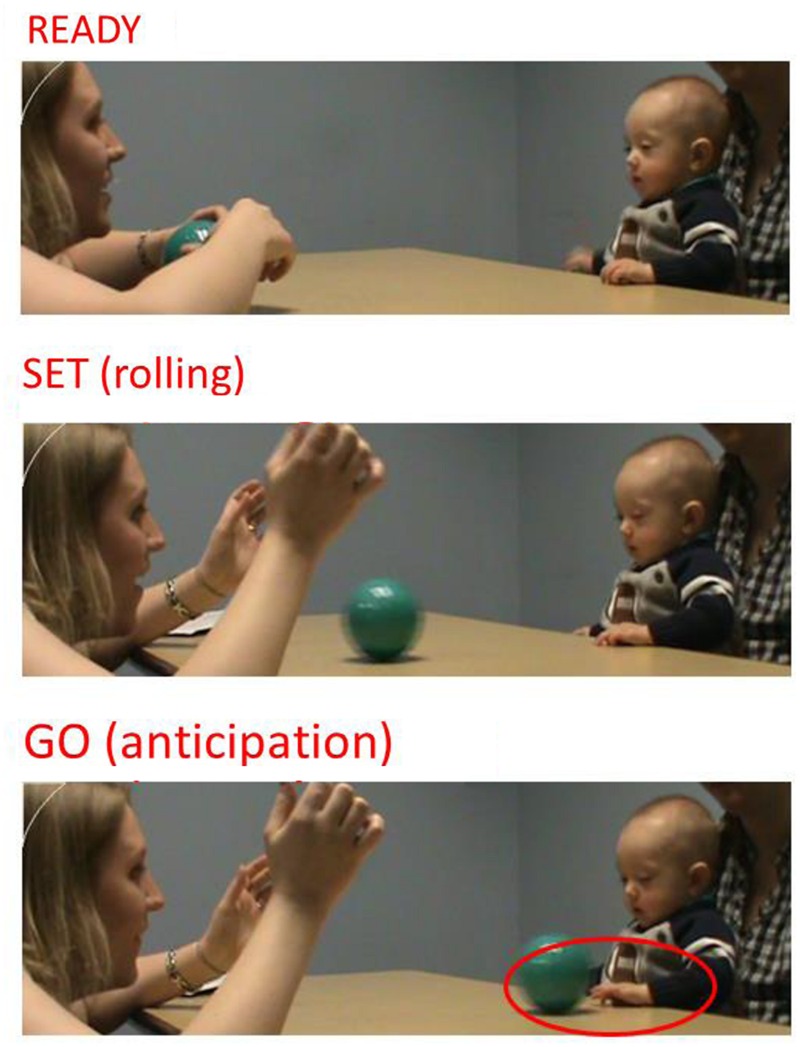
**Ready, Set, Go phases of the ball-rolling task**.

We coded archived videos of infants’ behavior during an experimental ball-rolling task initiated by an examiner. Infants were seated at a table on a caregiver’s lap opposite the examiner. Caregivers provided non-restrictive postural support at the hips, mid-trunk, or upper trunk, enabling infants to move their arms, hands, and trunk freely during the task. The task involved the use of a smooth-surface ball, approximately 5′ in diameter and easily clutched. Dependent variables were obtained across three phases (Ready, Set, Go; see **Figure 1**). The coding rubric is presented in **Table 2**. In the “Ready” phase, the examiner bounced the ball three times while smiling and looking at the child. During the “Set” phase, the examiner gently rolled the ball toward the child. The “Go” phase involved the child’s response to the approaching ball. At no time did the examiner provide verbal instructions to the infant.

**Table 2:** Coding schema for maturity of infant attention engagement and anticipatory response.

	Ready phase	Set phase	Go phase
Coder’s prompt for scoring	Just before the ball rolls to child, where is the child looking?	As the ball approaches, before contacting child’s body, where is the child looking?	Does the child move an arm or hand in anticipation of the approaching ball?
0	Child did not look at the ball or examiner	Child did not look at the ball during roll	No arm/hand movement toward the ball in anticipation
1	Delayed onset of gaze to the ball or examiner	As the ball approaches, child looks at least once to the examiner but not at the ball	Moves arm/hand in anticipation but no contact before ball hits body
2	Immediate gaze to ball **or** examiner	As ball approaches, child looks at the ball fleetingly (ball halfway across table before looking)	Moves arm/hand in anticipation, makes contact before ball hits body, but has to readjust hand position after contact to grasp the ball
3	Immediate gaze to ball **and** examiner	Child watches most of the ball’s trajectory toward him/herself (beginning when examiner releases the ball)	Moves arm/hand in anticipation with *open* hand, makes contact before ball hits body, and does not have to readjust hand position to grasp the ball

In the “Ready” and “Set” phases, coding focused on maturity of children’s attention engagement to relevant stimuli (examiner and ball). During the “Go” phase, maturity of children’s anticipatory motor response to the ball’s approach was coded, as indicated by whether and how children moved their hands toward the ball before it contacted their trunk. Videos were scored by two trained raters, who were blinded to risk status and achieved and maintained ≥90% inter-rater reliability using Cronbach’s Alpha measure for the Ready, Set and Go phases.

### Descriptive Measures

We used the Mullen Scales of Early Learning (MSEL; Mullen, 1995) Visual Reception, Gross Motor, and Fine Motor scales to assess non-verbal cognitive (visual pattern recognition, memory, and sequencing) and gross and fine motor skill, respectively, at ages 6 and 14 months. The MSEL is a standardized developmental test for children between ages 0–68 months. To investigate whether anticipation behavior at age 6 months was related to sensorimotor behavior at 14 months, a sensorimotor composite score was generated by summing age equivalency scores across the three afore-mentioned MSEL scales. Higher composite scores indicate more mature sensorimotor behavior.

The ADOS was administered at age 14 months to directly assess ASD symptoms, with all children receiving the Toddle Module (Lord et al., 2012) except for two children (1 HR) who received the ADOS-G Module 1 (Lord et al., 1999). The ADOS is a play-based standardized, gold standard assessment of ASD; higher ADOS Total scores represent greater impairment and greater concern for ASD in terms of social deficits.

As another measure of social engagement, we used an eye tracking paradigm to investigate infants’ attention to socially relevant stimuli (faces) at age 14 months (Libertus and Needham, 2011, 2014a,b). Pairs of faces and toys were presented side-by-side while infant eye gaze was recorded. A preference for faces was defined as the difference between the proportions of time spent looking at faces versus objects. Face preference values range from -100 to +100, with negative values indicating a visual preference for objects, 0 indicating no preference, and positive values indicating a visual preference for faces.

### Statistical Analyses

A logistic regression model was used to explore whether HR and LR infants could be distinguished based on their initiation of anticipatory action response during the ball-rolling activity. The primary independent variable (anticipation) was ordinal, but a number of continuous covariates were included in our model to account for potentially confounding sources of variability between the groups, including age and MSEL Gross Motor and Fine Motor performance at the time of examination. For the regression model, the dependent variable, ASD risk (HR, LR), was labeled according to the rule that *Y*_i_ = 0 if the i^th^ participant belonged to the LR group and *Y*_i_ = 1 if the i^th^ participant belonged to the HR group. Then,

$$\mathrm{logit}[P(Y_{i}=1)]=\beta_{0}+X_{i}\;\beta_{1}$$

where β_0_ is the intercept showing the average log odds of having high familial risk of ASD when all covariates are equal to zero, for each subject i; X_*i*_ is the vector of ball-rolling behavior scores and covariates added to account for potential confounders on the relationship between familial risk and ball-rolling task response. The exponentiated form of the log odds coefficients (*e*^β^) is reported for variables that were significant predictors of risk status along with the corresponding *p*-value.

Next to be examined was whether familial risk and early anticipation behavior are prognostic of later sensorimotor and ASD-related social functioning. In this analysis, familial risk for ASD and anticipation score at age 6 months served as independent variables. Stratifying subjects by risk (high/low) and anticipation at age 6 months (four levels: 0,1,2,3), two-way analyses of variance were used to test for between-group differences in ASD-related behaviors and sensorimotor skills observed at age 14 months. Additionally, *post hoc* simple main effect analyses and *t*-tests were performed to further investigate the influence of risk/anticipation combinations on outcome measures, with *p*-values adjusted for multiple comparisons using Bonferroni correction.

Finally, a logistic regression model was used to investigate whether early anticipation behavior was prognostic of later concern for ASD within the HR group. For this analysis, we grouped together all HR children who received an ADOS total scores indicating at least mild concern for ASD and compared them to HR infants whose ADOS total score indicated no concern for ASD.

## Results

### Association between Anticipation at Age 6 Months and Familial Risk for ASD

**FIGURE 2:**
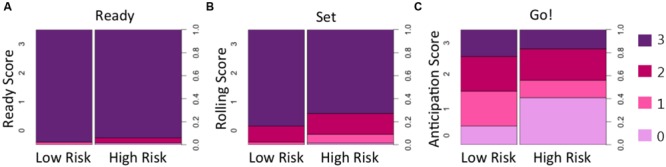
**Spine plots of behavioral responses during the three phases of the ball-rolling task.** The majority of children in HR and LR groups demonstrated context appropriate looking behavior by **(A)** looking immediately at the ball and/or examiner during the Ready phase (just prior to the examiner releasing the ball) and by **(B)** visually tracking the trajectory of the ball as it slowly rolled across the table toward them during the Set phase. However, the larger light purple area for the HR group in **(C)** indicates that HR infants were less likely to show a context appropriate motor response; they were more likely to fail to move in anticipation of catching the ball before the ball hit their bodies compared to LR infants.

Descriptive information about participants in each group (age, sex, MSEL Gross and Fine Motor scores, and total scores on the AOSI and ADOS) for HR and LR groups are presented in **Table 1**. Conditional distributions of the ball-rolling behavioral variables are presented in **Figure 2**. To explore whether HR and LR infants could be distinguished based on their anticipatory action response during the ball-rolling activity, we constructed a logistic regression model, which included a number of covariates to account for potentially confounding sources of variability between familial risk groups. After plotting the conditional distributions of the ball-rolling behavioral variables, Ready and Set phase data were excluded as covariates because nearly all children performed at ceiling during these phases by (a) immediately directing their attention to the ball and/or examiner prior to the roll and (b) visually tracking the ball for most of its trajectory toward them. The proportion of infants who failed to immediately direct their attention to the ball and/or examiner and visually track the ball for most of its trajectory did not differ significantly between familial risk groups (ready: X2 = 0.19, *p* = 0.66; roll: X2 = 0.10, *p* = 0.75). Including age on the day of the ball-rolling experiment, MSEL Gross and Fine Motor scores, and anticipation score as predictors of familial risk, our model as a whole fit significantly better than the intercept only model (X2 = 17.97, df = 6, *p* = 0.006, McFadden pseudo-*R*^2^= 0.212). **Table 3** lists odds ratios, *z*-statistics, and the associated *p*-value for each predictor. As can be seen from **Table 3**, we detected a marginal between-group age difference. On average, HR infants were slightly older than LR infants at the time of the targeted age 6-month examination, and a 1-month increase in age at the time of examination was associated with decreased odds of belonging to the LR group by a factor of 0.40 (*p* = 0.076). This age difference could have afforded the HR group a slight performance advantage.

**Table 3:** Predictors of familial risk for ASD at age 6 months.

Predictor	Odds ratio	*z*-Statistic	*p*-Value
lAge	0.40	-1.78	0.076
lMSEL Gross Motor	1.18	0.83	0.407
lMSEL Fine Motor	1.62	2.32	0.020
lAnticipation 0–1	5.78	2.68	0.007
lAnticipation 0–2	3.32	1.93	0.053
lAnticipation 0–3	4.57	2.28	0.023

*Positive odds indicate that an increase in the predictor variable (e.g., going from an anticipation score of 0–1) results in an increase in the odds of a participant belonging to the low risk group (versus belonging to the high risk group). The most common score was used as the reference level (0).*

The logistic regression model revealed a significant effect of MSEL Fine Motor score on familial risk (HR vs. LR group). Analysis of MSEL motor scales revealed that, on average, HR infants displayed less mature fine motor skills; a one unit increase in MSEL Fine Motor score increased the odds of belonging to the LR group by a factor of 1.62 (*p* = 0.020). In contrast, 6-month-old MSEL Gross Motor scores were not predictive of familial risk (*p* = 0.407).

After controlling for age and fine and gross motor skills (based on MSEL Fine and Gross Motor scores), a main effect of anticipation on familial risk group (X2 = 8.4, df = 3, *p* = 0.039) was identified during the “Go” phase of the task. Regardless of the maturity of the movement, moving a hand/arm in anticipation of the approaching ball (score of 1–3; see **Table 2**) was associated with decreased odds of belonging to the HR group. Displaying no movement in anticipation of catching the ball increased the odds of belonging to the HR group by a factor of 5.78 (*p* = 0.007) compared to displaying a delayed hand/arm movement (score of 1), by a factor of 3.32 compared to successfully anticipating the ball but having to readjust hand position after making initial contact (score of 2) (*p* = 0.053), and by a factor of 4.57 compared to successfully anticipating the ball with the proper hand orientation to grab the ball (score of 3) (*p* = 0.023). In other words, HR infants were less likely than LR infants to initiate action in anticipation of the approaching ball (**Figure 2C**).

### Association between Anticipation at Age 6 Months and Social and Sensorimotor Functioning at Age 14 Months

**FIGURE 3:**
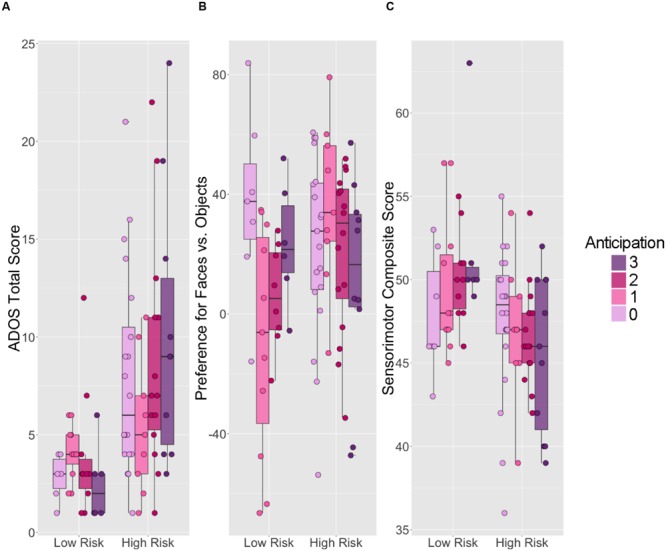
**Six-month anticipation behavior versus 14-month social and sensorimotor outcomes for children at low and high familial risk for autism.** Boxplots displaying **(A)** ADOS Total Score, **(B)** preference for faces compared to objects, and **(C)** MSEL sensorimotor composite scores. Within each panel, scores for LR children are on the left; scores for HR children are on the right. The upper and lower “hinges” correspond to the first and third quartiles of the distribution for each risk group at each level of 6-month anticipation. Higher **ADOS Total scores** represent worse performance. Face preference values range from -100 to 100, with negative values indicating a visual preference for objects, 0 indicating no preference, and positive values indicating a visual preference for faces. Sensorimotor composite scores were calculated by summing three relevant age-equivalent subscale scores from the Mullen Scales of Early Learning (MSEL): Visual Reception, Gross and Fine Motor. Higher sensorimotor composite scores represent better performance.

A two-way ANOVA was conducted to examine relationships among familial risk for ASD (two levels), anticipation behavior at age 6 months (four levels) and total ADOS score at age 14 months. There was a statistically significant main effect of familial risk on total ADOS score [*F*_(3,78)_ = 18.656, *p* < 0.001], with the HR group, on average, exhibiting a higher total score on the ADOS (mean [SD]: 8.15 [5.6]) and more behaviors consistent with ASD than the LR group (3.52 [2.2]). However, there was no significant interaction between familial risk for ASD and anticipation at age 6 months on 14-month total ADOS score [*F*_(3,78)_ = 1.40, *p* = 0.25] (**Figure 3A**). **Table 4** displays 6-month-old anticipatory score counts for the HR group stratified by ASD concern at 14 months of age. Excluding the one HR subject who received the ADOS-G from our logistic regression model of 14-month concern for ASD, 17 children in the HR group scored high enough on the ADOS-T to warrant at least mild concern for ASD. After controlling for age and fine and gross motor skills at age 6 months within the HR group, we did not observe a main effect of 6-month anticipation on 14-month concern for ASD (X2 = 0.79, df = 3, *p* = 0.85).

**Table 4:** Six-month anticipatory behavior in the High Risk group stratified by concern for ASD at 14-month-old.

6-month Anticipation	HR at 14-month-old	
	**No ASD Concern**	**At least Mild ASD Concern**
	***n* = 36**	***n* = 17**
	
0	14 (38.8%)	6 (35.2%)
1	7 (19.4%)	2 (11.7%)
2	9 (25%)	5 (29.4%)
3	6 (16.7%)	4 (23.6%)

*The one HR subject who received the ADOS-G instead of the ADOS-T at the 14-month-old time point was excluded from this analysis.*

**Figure 3B** displays age 14-month preference for looking at faces stratified by ASD risk group and age 6 month anticipation. We observed a significant interaction between familial risk for ASD and anticipation at age 6 months on our outcome of interest at age 14 months [F_(3,78)_ = 4.375, *p* = 0.007]. Simple main effects analysis revealed no significant difference between 14-month preference for faces in HR and LR children who did not move in anticipation of catching the ball at age 6 months (*t* = 1.00, df = 3, *p* = 0.34). However, comparison of 14-month face preference behavior of HR and LR children with 6-month anticipation scores of 1 (delayed onset of anticipatory movement) revealed that the HR children exhibited a stronger preference for faces than LR children (*t* = 3.099, df = 17.8, *p* = 0.006); on average, HR children who exhibited a delayed anticipatory response to the ball spent 36.6% more time looking at faces than at objects, while LR children who exhibited a delayed onset of anticipatory response to the ball actually spent 8.9% more time looking at objects than at faces. In addition, we observed a simple main effect of anticipation on preference for faces within the LR group [*F*_(3,28)_ = 3.373, *p* = 0.02]. Follow-up *t*-tests suggested that LR children who did not move in anticipation of catching the ball exhibited much stronger preference for faces than LR children who exhibited a late (delayed) anticipatory response (*p* = 0.009). Such relationships between anticipation and preference for faces were not observed in the HR group [*F*_(3,50)_ = 1.14 = 39, *p* = 0.342], likely because of the high level of face-looking in the HR infants who showed delayed activation of anticipatory response.

**Figure 3C** displays 14-month sensorimotor composite scores stratified by ASD risk group and 6-month-old anticipation. We observed a statistically significant main effect of familial risk for ASD on the study-defined sensorimotor composite score at age 14 months [*F*_(3,78)_ = 12.4, *p* < 0.001]; on average, LR children demonstrated higher sensorimotor composite scores (49.9 [4.00]) than HR children (46.7 [4.03]). However, the interaction between familial risk for ASD and anticipation at age 6 months on sensorimotor behavior at age 14 months was not statistically significant [*F*_(3,78)_ = 1.67, *p* = 0.18].

## Discussion

### Overview of Findings

The aim of this study was to examine whether early ASD risk indicators are detectable in mid infancy, particularly perception-guided predictive action, or initiating anticipatory hand/arm action in response to a visibly approaching object during dyadic interpersonal engagement. To our knowledge, this is the first such study in infants at heightened familial risk for ASD. Six-month-old infants at HR and LR for ASD engaged in a dynamic, interactive dyadic ball-rolling activity with an examiner. No group differences were observed in the infants’ visual attention before (‘Ready’ phase) or during (‘Set’ phase) ball rolling; both groups demonstrated context appropriate looking behavior, indicating engagement in the activity. However, as hypothesized, HR infants displayed decreased perception-guided predictive action in response to the approaching ball compared to LR infants. This finding did not appear to be driven by differences in motor ability between risk groups at 6 months of age. No relationship was identified in the HR group between 6-month anticipatory action response and 14-month sensorimotor functioning or degree of autism symptomatology, an age window within which many children who will later be diagnosed with ASD remain within the ASD prodromal phase. Surprisingly, HR infants with emerging anticipatory response (score of 1) at age 6 months exhibited greater face preference performance at age 14 months compared to LR infants at the same level of anticipatory response maturity at age 6 months.

### Anticipatory Action Responses at Age 6 Months

To successfully anticipate catching a moving object, infants must rapidly incorporate visual information about the size and speed of the ball into their motor plan. Despite their attention to the approaching ball, a subgroup of HR infants completely failed to translate visual information into a successful motor response. Our finding of lower anticipatory action response to the approaching ball in the HR group indicates that the deficit in visual-motor coupling and anticipatory responses previously identified in older children with ASD, even those without intellectual developmental delay, have roots in infancy (Mostofsky et al., 2000; Schmitz et al., 2003; Martineau et al., 2004; Cattaneo et al., 2007; Whyatt and Craig, 2013; Ament et al., 2015; Sharer et al., 2015). Our finding extends that reported by Brisson et al. (2012) whose retrospective study identified a deficit in anticipatory mouth opening in 4- to 6-month-olds later diagnosed with ASD when a spoon approached during feeding. Similarly, Cattaneo et al. (2007) found that children with ASD, unlike those with typical development, failed to show anticipatory mylohyoid activation during observation or execution of grasping an object to eat. A diminished capacity to move in an anticipatory manner at such an early age could reflect a deficit in the brain’s ability to construct an internal model relating visual feedback with motor output. Such internal action models are thought to be critical not only to predict the sensory consequences of self-generated motor commands but also to interpret the actions of others.

In our study, the failure of the HR infants’ initiation of hand movement to ‘catch’ the ball rolled toward them by a social partner could be due to lack of attention, lack of motivation to stop and explore the ball, motor impairment (Bhat et al., 2011), diminished motion detection or direction capabilities, failure to comprehend the social contingency of the dyadic game (Nadel, 2002), impaired coupling of visual and temporal information to guide and adapt movement (Ament et al., 2015), or a motor planning deficit resulting in poor timing of movement execution (Forti et al., 2011). In the present study, less mature fine motor skills were identified in the HR than LR infants. However, this immaturity cannot fully account for our finding of anticipatory deficit in HR infants as motor skill level was controlled for in the analyses. Attention and motivation are not viable explanations either, as all infants in both groups were highly attentive to the task and explored the ball after it had been rolled to them (whether or not they demonstrated anticipatory action response). Existing literature suggests that basic motion detection and direction perception thresholds are largely unimpaired in ASD (Mottron et al., 2006) and are not likely to blame for the reduction in anticipatory responses in the HR group. Regardless of the specific underlying mechanism, a lack of anticipatory response during infancy could contribute to a short- or longer-term developmental cascade in which infants’ anticipatory failure thwarts the unfolding dynamic exchange with the social partner and diminishes the quality or quantity of social input, which could thereby attenuate social outcomes (Hofer et al., 2008). Early failure of anticipatory action response also could impede infants’ learning about action contingencies, which plays a role in the development of action control (Hauf et al., 2004) and could impact a host of later-emerging motor-related social functions such as imitation and joint attention.

### Relationship between 6-Month Anticipatory Responses and 14-Month Sensorimotor and Social Behavior

Although a subgroup of HR infants completely failed to translate visual information into a successful motor response at age 6 months, this failure was not associated with an increased likelihood of showing more signs of ASD at age 14 months, as measured by total score on the ADOS. This finding was surprising given others’ findings that visual-motor integration deficits are associated with social deficits (Haswell et al., 2009). Perhaps our negative finding is related to the fact that ASD symptoms are just emerging in most children with ASD during the second year of life (Ozonoff et al., 2010; Landa et al., 2013), and about half of children later diagnosed with ASD remain prodromal at age 14 months (Landa et al., 2007, 2013). Perhaps anticipatory behavior at age 6 months will predict ASD at a slightly older age, subsequent to the ASD prodromal phase. Reduced anticipatory response at age 6 months also was not associated with worse sensorimotor performance at age 14 months, as measured by our study-defined sensorimotor composite score. However, we did observe a significant interaction between 6-month anticipation and familial risk for ASD on 14-month attention to faces.

We found that, in the HR group only, emerging anticipatory response (as opposed to absent or acquired) at age 6 months was associated with increased levels of face-looking at age 14 months. High levels of face looking actually may not be typical at age 14 months. In a recent study of face looking in infants from 3 to 11 months of age and adults using an eye tracking task identical to the one used herein, Libertus and Needham (2014a) identified an inverted U shaped curve over time such that lowest levels of face-looking occurred at 3 and 11 months of age; no face preference was identified in the adults. Indeed, our finding of stronger face preference at age 14 months in LR infants who failed to move in anticipation of catching the ball than in those with emerging anticipatory response may indicate a relationship between early observable immaturity in anticipatory behavior and a later atypicality in proportion of time spent looking at faces. This is echoed by the LR infants who exhibited no anticipatory response, as they demonstrated more preference for faces compared to anticipating LR infants. The relationship between early anticipatory behavior and later face preference requires further investigation in children at low and HR for ASD.

The findings discussed above extend our (Landa and Garrett-Mayer, 2006; Bhat et al., 2012; Flanagan et al., 2012; Libertus and Landa, 2013; Libertus et al., 2014) and others’ (e.g., Iverson and Wozniak, 2007) reports of motor-related delays in HR infants, others’ reports of anticipatory deficits in ASD (e.g., Cattaneo et al., 2007; Brisson et al., 2012), and of visuo-motor abnormalities in ASD (e.g., Sharer et al., 2015), even a report of ASD-specific abnormality in ball catching in high-functioning children with ASD (Ament et al., 2015). Also, these findings are consistent with the hypothesis of predictive impairment in autism which attempts to explain autism as a general impairment in information processing (Sinha et al., 2014) and predicts that motor anticipation will be reduced in individuals with ASD.

### Limitations

The greatest limitations of the present study include sample size and the absence of information regarding diagnostic outcomes. Although the sample size was sufficient to detect group differences at age 6 months and moderately distal (8 months later) relationships between infant action anticipation and attention to faces in toddlerhood, ideally we would like to probe whether infant action anticipation is associated with concern for ASD at a developmental stage that is more commonly used for diagnosis (24- or 36 months of age). We are not yet able to discern the implications of deficits in perception-guided predictive action at age 6 months for the emergence of the autism phenotype.

### Future Directions

Increasingly, emerging evidence highlights the importance of early action experiences for short and longer-term motor, cognitive, and social outcomes. Yet there is much to learn, particularly with regard to when and how developmental processes are altered in infants at HR for ASD. Research is needed to define the relationship between action control, visual attention, motor skill, action anticipation, contingency learning, imitation and joint attention concurrently and over time during the first 3 years of life. In particular, mechanisms that support action anticipation in HR infants and how these may differ from those in LR infants require definition. The decoupling of early execution of anticipatory action and social functioning 8 months later in HR infants should be further investigated and requires replication. In addition, research focused on the impact of early dyadic, reciprocal action routines and self-generated action experiences in HR infants is needed to determine potential for improving later social functioning; promise of such effects are emerging (Libertus and Landa, 2014; Libertus and Needham, 2014b). Such research is important because the abilities that afford execution of anticipatory action involve the integration of visual information with motor performance, which is needed in the development of internal action models. Such action models likely are relevant to development of motor skills central to social development such as imitation, production of interpretable and well-timed interpersonally synchronous actions, as well as for the understanding of others’ action intentions. Ultimately, the goal is to detect developmental vulnerabilities as early in life as possible in infants at HR for ASD and to optimize their outcomes.

## Author Contributions

RL: study design, task development, coding schema development, conceptualizing the study, writing the manuscript. JH and MBN: coding schema refinement, coding the data, conducting analyses, writing the manuscript.

## Conflict of Interest Statement

The authors declare that the research was conducted in the absence of any commercial or financial relationships that could be construed as a potential conflict of interest.
